# Study on the Vibration Characteristics of the Telescope T80 in the Javalambre Astrophysical Observatory (JAO) Aimed at Detecting Invalid Images

**DOI:** 10.3390/s20226523

**Published:** 2020-11-15

**Authors:** Fernando Arranz Martínez, Raúl Martín Ferrer, Guillermo Palacios-Navarro, Pedro Ramos Lorente

**Affiliations:** 1 Department of Computer Science and Systems Engineering, University of Zaragoza, 44003 Teruel, Spain; farranz@unizar.es (F.A.M.); ramar@unizar.es (R.M.F.); 2 Department of Electronic Engineering and Communications, University of Zaragoza, 44003 Teruel, Spain; pramos@unizar.es

**Keywords:** accelerometer, vibration signature, telescope vibration, spectral estimation, monitoring

## Abstract

The location of large telescopes, generally far from the data processing centers, represents a logistical problem for the supervision of the capture of images. In this work, we carried out a preliminary study of the vibration signature of the T80 telescope at the Javalambre Astrophysical Observatory (JAO). The study analyzed the process of calculating the displacement that occurs because of the vibration in each of the frequencies in the range of interest. We analyzed the problems associated with very low frequencies by means of simulation, finding the most critical vibrations below 20 Hz, since they are the ones that generate greater displacements. The work also relates previous studies based on simulation with the real measurements of the vibration of the telescope taken remotely when it is subjected to different positioning movements (right ascension and/or declination) or when it performs movement actions such as those related to filter trays or mirror cover. The obtained results allow us to design a remote alarm system to detect invalid images (taken with excess vibration).

## 1. Introduction

In professional astronomy, mapping the universe requires high levels of precision, so the stabilization of the instrumentation is of paramount importance in ensuring the quality in image capture. The design and commission of telescopes require that the tracking actuators do not transfer their disturbances, translated into vibrations, to the quality of the image taken. This is why the professional telescope validation process requires that the sum of the vibrations of all the actuators be below the maximum value of the vibration allowed by the instrumentation.

### 1.1. Vibration in Large Structures

In large civil structures, it is essential to detect vibration frequencies as an alarm against their structural resonance limit. We found examples in the supervision of large bridges [1], for example, in the study conducted by Abdel-Ghaffar and Scanlan [2], the structure of the Golden Gate was supervised in order to establish those frequencies produced in the environment that can make the structure come into resonance, thus leading to a possible collapse. The importance of controlling, monitoring, and predicting the behavior of bridges begins with studies in controlled environments [3] in which tests are carried out to verify the behavior of the future structure according to the environmental conditions measured at the location. Buildings are other large structures that also require monitoring [4]. The wind force can produce vibrations that can cause them to collapse if they come into resonance. In older buildings such as the Empire State Building [5], vibration monitoring is based on the supervision of risks due to vibrations that damage the structure. These risks may be based on weather reasons such as the wind, earthquakes, or artificial phenomena such as traffic. We can also find more examples in underground railway infrastructures [6]. In this case, the authors used the signals in the time domain to study the accelerations produced in a railway tunnel. They implemented vibration absorption systems using springs, improving the level of vibration transmitted by the ground and observed that the accelerations were below the limits allowed in the design. Tunnels are also large structures that require analysis of the behavior of vibration throughout the structure in order to estimate its stability. In their study, Tian et al. [7] determined different safety zones in a tunnel as the vibration was decreased along the measurement axis.

### 1.2. Vibration in Telescopes

Professional telescopes are large structures that require supervision and the control of vibrations during their operation. The stability of the instrumentation during image capture requires monitoring all accelerations that may occur due to the stability of the terrain, weather conditions, or movement of the actuators. Vibration measurement is used to ensure that its values do not exceed the maximum allowed by design and does not affect the image quality. Different vibration damping mechanisms are used for this purpose.

In this field, numerous research works have focused on the study of vibrations. Many of them have focused their objectives on introducing control algorithms in the regulation loops of the actuators. In the study carried out at the Subaru Telescope [8], the authors found that the captured images had elongation in the azimuth and elevation. They concluded that frequencies of a few hertz affected the camera and also related the movements and the value of the acceleration with the actuator that caused it. The study determined that some of the vibrations measured could not be solved mechanically and that it was necessary to adjust the filters of the control loops.

On the other hand, in the study carried out at the MMT Observatory [9], the accelerometers were placed on the secondary mirror. The measurements served the rest of the positioning instrumentation to feedback information into the control loop and achieve greater stability. Along this line, the study carried out in the E-ELT/MICADO Telescope [10] took into account the disturbances produced by the atmosphere and those produced mechanically by the structure that affect image quality. To do this, they implemented control loops in a single regulatory system that compensates for each of them. The filters they used turned out to be good at high frequencies but delayed the reconstruction, which led to a reformulation of the solution based on adaptive resonators. The study by Keck et al. focused on long telescopes [11] and the authors intervened in the actuator control loop to improve stability in the optical plane. Using a single accelerometer, they studied the vibrations produced by the atmosphere and those due to the structure of the telescope. They established a control loop for the vibrations of the structure and for the vibrations produced by the atmosphere, the result of which was an improvement in the precision of positioning through adaptive resonators.

Other studies have opted for the use of seismological accelerometers and resonant structures that allow for quantifying the disturbances produced at very low frequencies (up to 4 Hz). This is the case of the research carried out at the Daniel K. Inouye Solar Telescope [12], where the authors managed to establish coherence between different points of the upper and lower part of the assembly of more than 80%, and therefore found the sources of the forces that caused it. The research carried out at the William Herschel Telescope (WHT), Big Telescope Azimuthal (BTA), and Canada–France–Hawaii Telescope (CFHT) [13] focused their efforts on analyzing the vibrations produced in the telescope and their effect on the acquisition of images. To do this, they measured very weak movements below the positioning instrumentation with accelerometers, which allowed them to verify that the positioning was correct and to establish the bases of the improvement in other similar telescopes.

At the Thirty Meter Telescope (TMT), researchers Thompson et al. [14] focused on the study of the azimuth motor gear on the *A*–*Z* axis. To do so, they estimated the forces produced in the gear of the direct motor, taking into account the misalignment in the installation and the spatial compensations. They used several complementary methods that consisted of calculating the gear forces and compensating the telescope. They determined that the movement that most influenced the image was elevation 80° and the most problematic frequencies were found in the range of 5–20 Hz.

The study of Trigona, Andró, and Baglio [15] was carried out at the telescope installed at the “Serra la Nave” Observatory. This showed the effect that the movements of the telescope had on the production of vibrations at different points of the structure. To demonstrate this, they used a 3D setup formed by a triaxial structure of three accelerometers located at different parts of the telescope. The movements recorded were measured in the time domain and transformed to the frequency domain in an interval from 0 Hz to 1 kHz, since this is the range where the most dangerous acceleration levels are concentrated. After carrying out different movements, they determined that the horizontal movement, together with the location of the accelerometers at the end of the telescope, gave an acceleration peak at the frequency of 5.5 Hz with a maximum acceleration of 0.1 *g*.

The previous studies have had as a fundamental objective the detection of the origin of the vibrations produced in order to use actuators that compensate them, or once identified, are able to act a posteriori on the images taken to solve the derived problems, either mechanical problems [11] or quality of image related problems [10]. To meet this objective, a specific technology can be used, which we list below. While the previous publications focused on measuring the vibration in the actuators or at critical points of the structure using arcseconds as a displacement unit [15], we measured the displacements in the CCD in micrometers. The purpose was to obtain the displacement of the pixel with respect to the point of light to be captured. In this way, it will be possible to estimate the number of photons that are captured in the pixel itself and in neighboring pixels. Furthermore, the so developed monitoring remote system overcomes the access problems related to the location of large telescopes, which are normally located in high mountain areas where access is complicated due to both the orography and the weather conditions. This fact adds a notable improvement with respect to the studies found in the literature.

### 1.3. Required Technology

The precision required by the capture instrumentation makes professional telescopes look for solutions to mitigate the loss of quality in the image due to vibration [8,9,12,16]. In mapping astronomy, it is not only a question of reducing the effects of unwanted movements, but of quantifying the displacements produced in the charge coupled device (CCD), thus estimating the loss of light in the pixel. The vibration quantification methods vary, from 3D vision [17], piezoelectric accelerometers [8,9], electrostatic resonators [18], or fiber optic accelerometers with Fabry–Perot technology [19], among others. Other studies have chosen the use of low frequency accelerometers of the seismological type and resonant structures. These systems make it possible to quantify—at very low frequencies (up to 4 Hz)—the disturbances produced in adaptive optics with one input and one output control loop (SISO). This was the case in the research carried out in the Daniel K. Inouye Solar Telescope [12], where, in addition, coherence analysis was used to validate the results taken at different points.

Piezoelectric sensors are widely used in vibration-based research. These sensors have good sensitivity and measurement ranges that reach very low frequencies (close to 0 Hz). On the other hand, they are very sensitive to electromagnetic fields, which can lead to the coupling of unwanted signals such as the electrical network. The elimination of these undesirable frequency couplings is solvable by filtering software techniques [20,21,22].

The objective of this article was to establish the basis for the design of an excess vibration detection system in the T80 telescope. The system should warn of excessive displacements in the CCD of the T80Cam camera of the T80 telescope. Through signal processing, the frequencies likely to generate displacements that result in blurry images not compensated by optical actuators (AO) will be determined.

The experiment presented in this article was developed at the facilities of the Javalambre Astrophysical Observatory (JAO) [23,24,25], located in the “Pico del Buitre” (Arcos de las Salinas, Teruel), which is managed by the Aragon Center for Physics of the Cosmos (CEFCA). Verification tests were carried out in a controlled environment installed in the Active Noise Control laboratory (A.N.C.) at the Teruel Polytechnic School of Engineering (Teruel, Spain).

The rest of this article is organized as follows. Section 2 focuses on the description of the materials and methods used as well as the work environment. Section 3 introduces the theoretical foundations our study is based on, whereas Section 4 presents the results obtained after the experiments were carried out both in a controlled environment and in the facilities where the T80 telescope is located. Finally, Section 5 presents the main conclusions of the work as well as future lines of research.

## 2. Materials and Methods

### 2.1. Instrumentation Used and Working Environment

The equipment used consisted of three Wilcoxon (Frederick, MD, USA) piezoelectric accelerometers (731A model), with dynamic vibration output (IEPE) and sensitivity 10 V/*g* with working frequencies between 0.05 Hz and 450 Hz. The weight of each accelerometer was 760 g and had dimensions of 6.22 cm in diameter by 7.34 cm in height. This type of accelerometer has previously been used to measure low frequencies (up to 0.1 Hz) [26] and to monitor civil structures [18], radio telescopes [27], and telescopes [8,9,10]. The accelerometers were connected to a data acquisition module composed of four input channels. The data acquisition module was a National Instruments (NI-cDAQ-9234 model, IEPE type) with a Delta-Sigma ADC with 24 bits per channel and a range of 51.2 kS/s. The module was connected to a chassis for eight input/output National Instruments (Austin, TX, USA) NI-cDAQ-9148 modules, a power supply by external DC voltage source, and an Ethernet communication port between 10 MHz and 100 MHz. Table 1, Table 2 and Table 3 show the main characteristics of the used equipment.

#### 2.1.1. Controlled Environment

The above equipment was installed in the Active Noise Control laboratory of the EUPT to carry out the experiments in a controlled environment. In this testing phase, we carried out the task of designing and debugging the processes for measuring and dynamic representation of the controlled vibrations that were generated in the laboratory. The general objective of this phase is to master data collection in a controlled environment so that we can solve later eventual setbacks effectively in the subsequent assembly phase of the measurement system in the JAO. The time of access to the observatory facilities is very limited and we sought maximum efficiency in the real work context, where we cannot afford any unjustified delay in the activities planned for each work session.

#### 2.1.2. Experimental Environment

**Figure 1:**
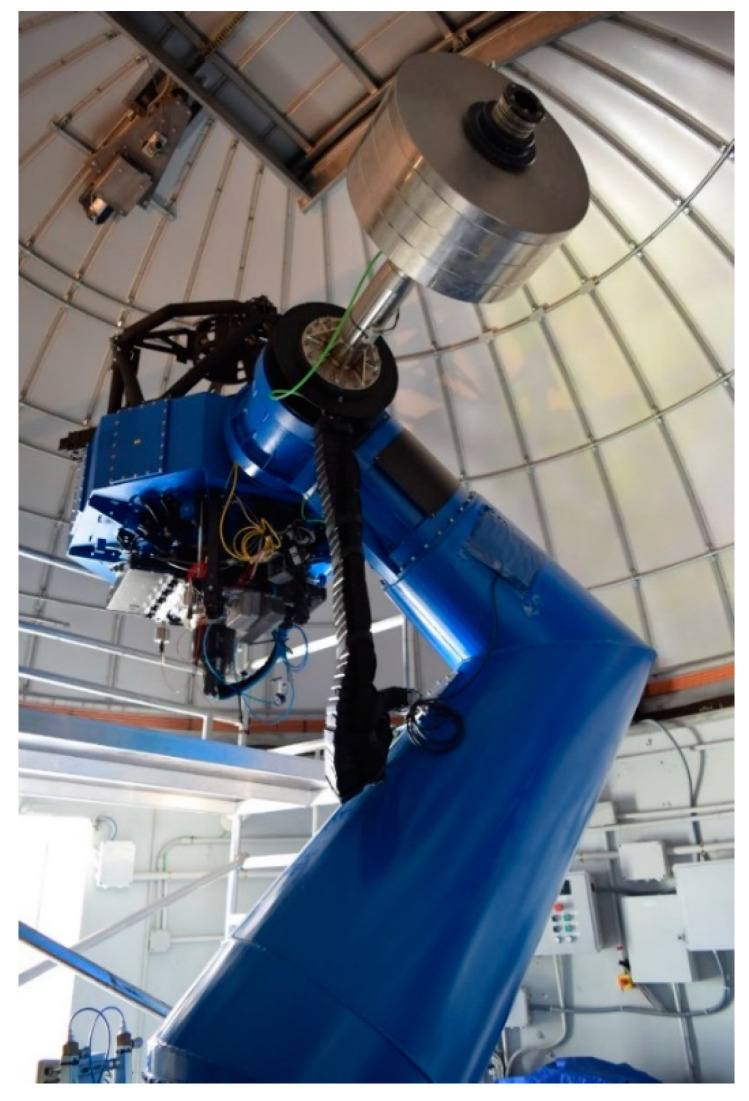
Detail of the T80 telescope.

**Figure 2:**
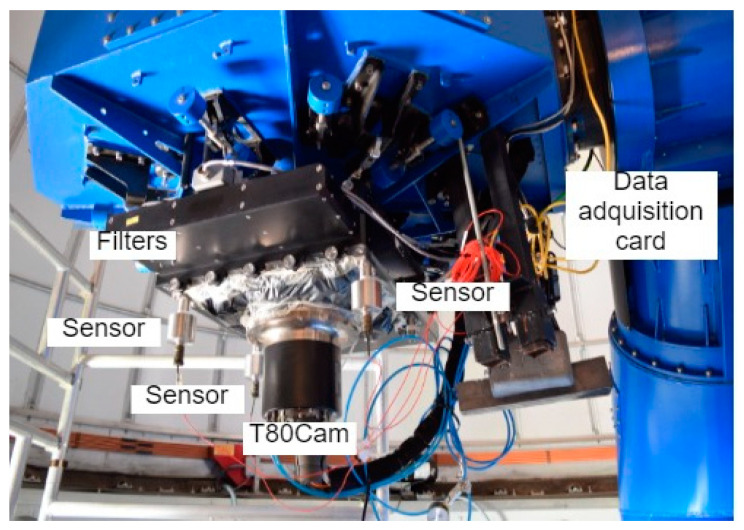
Detail of the T80Cam camera, filters, accelerometers, and data acquisition equipment.

Once the preliminary study was carried out in the controlled environment, the hardware was moved to the JAO premises and installed on the T80 telescope. The T80 telescope has an 80 cm Ritchey–Chrétien mount with a 2° field of view. Its design is focused on supporting the realization of large maps, being provided with technology that compensates for temperature changes and/or mechanical bending in different parts of the telescope. The set has a weight of 2500 kg and is capable of supporting instrumentation of up to 80 kg. The current instrumentation is the T80Cam with a 9.2 k × 9.2 k pixel CCD and a pixel size of 10 µm, along with a set of 12 filters on two rotating trays [28]. Figure 1 shows the general appearance of the T80 telescope, while Figure 2 shows the detail of the T80Cam camera, filters, accelerometers, and data acquisition equipment. The accelerometers were installed on the back of the filters, just above the T80Cam and at the same height as the CCD. Due to the mechanical and dimensional characteristics of the T80Cam, it was not possible to place them on it. The three accelerometers were placed perpendicular to the XY plane in the direction of the *Z* axis with respect to the CCD. It was checked that the weight of the accelerometers did not show relevant incidences in the compensation of the weight of the camera. The use of a triaxial structure can generate mismatches in the instrumentation due to the high weight of three accelerometers in a single point.

The T80 telescope has two movements perpendicular to each other: horizontal movement (or declination) and vertical movement (or straight ascension). It also has two speeds of movement, on one hand, the “tracking” movement is used to go to the position of the camera to the point in the universe to be measured. This movement is characterized by high speeds. The vibrations do not affect the whole telescope except with values higher than the technical specifications of the most sensitive element; in this case, the T80Cam, which had a value not higher than 1 *g*. On the other hand, the movement of “slewing”, through which the camera moves, compensating the movement of the Earth to keep track during the exposure time. It is in this case that stability and precision must be at the maximum to avoid vibrations.

### 2.2. Methods

**Figure 3:**
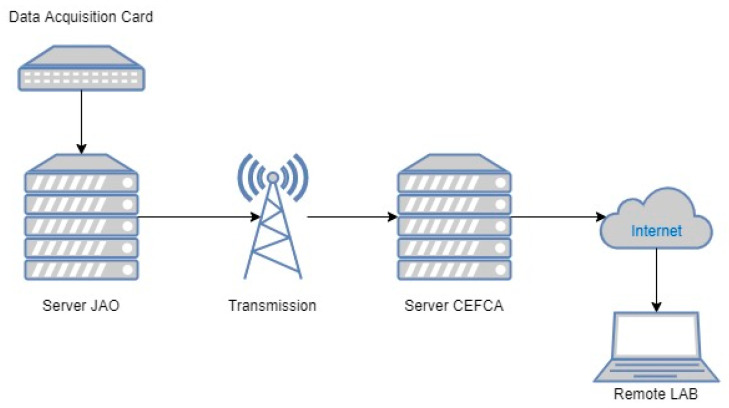
Communication scheme between JAO-CEFCA and a remote laboratory.

Two applications were used to operate the system, one for communication between the data acquisition card and the computer, and the second one for data processing. Data acquisition was carried out using a software platform that the manufacturer supplies for communication between the computer and the acquisition card. For the information treatment and signal processing, MATLAB (Natick, MA, USA) version 2018 was used with the “Analog input recorder” toolbox. With regard to the communication system, the data from the accelerometers located in the JAO were collected on a local server and transmitted to the server at CEFCA headquarters. The data can then be available through a computer from any remote laboratory. Figure 3 describes the communication scheme.

Data acquisition begins with the remote connection of the CEFCA servers and the application that accesses the data acquisition card. Subsequently, the MATLAB 2018 work platform is started together with the Toolbox. Data processing with MATLAB begins with establishing the route with the data acquisition card. Only three channels were used, reserving the fourth channel for future experiments.

## 3. Theoretical Foundations

One of the main objectives of this work was to determine the displacements caused by vibration in the plane in which images are taken. The comparison between this displacement and the maximum allowed, given by the sub-pixel distance, is the basis of the system that must warn us that some images have been taken in conditions of excess vibration. With the following theoretical analysis, we intend to estimate the acceleration limit values so that they do not generate a displacement greater than the sub-pixel distance.

The theoretical foundations of our system were based on a simplified model in which the vibration—the measured acceleration—is a pure frequency. This is a good approximation of what we frequently find in acceleration measurements taken in the neighborhood of the telescope camera. White [29] performed a similar simplified work for the motion of a mass hanging from a spring, approximating the motion as a simple harmonic.

The principle is as follows: once the maximum admissible displacement is set, given by the subpixel distance, the maximum acceleration is estimated at each frequency. When this maximum value is exceeded, the quality of the image taken is considered poor and the image can be rejected or subjected to post-processing treatment (if appropriate). This idea allowed us to develop an alarm that warned us whenever an acceleration component could cause a displacement above the admissible threshold. It must be taken into account that the displacement is measured at the point where the acceleration is measured, which are the points closest to the CCD.

We start from the acceleration, which is what we measure with the accelerometer:

(1) a=A cos(ωt) 

$$A\;\mathrm{in}\;\frac{m}{s^{2}}.$$

By double integration and assuming zero integration constants, a displacement *d* is obtained

(2) $$d=\left(-A/\omega^{2}\right)\;cos\left(\omega t\right).$$

The maximum displacement is therefore: $\frac{A}{\omega^{2}}$. 

If, on the other hand, we choose a limit value (*dsubpixel*) equal to 5 μm, which is the maximum size from which image distortion will occur [30],

(3) $$\frac{A}{\omega^{2}}<dsubpixel.$$

The measurement made is *Ag*, the amplitude of the acceleration in *g*, while the equation uses the acceleration of gravity, so it is necessary to make the conversion according to$\;1\;g=9.8\;m/s^{2}$. Solving for:

(4) $$A_{g}<\frac{{\left(2\pi f\right)}^{2}\;dsubpixel}{9.8}\;.$$

The process to determine if a signal exceeds the subpixel limit value starts from the transformation of the signal a(n) to in its frequency representation through the fast Fourier transform (FFT). Let |Af(k)| be the module of the FFT of size *N* of the sequence a(n). Instead of representing this discrete vector of samples |Af(k)| in the frequency domain, we make a representation only in positive frequencies (single-sided) of the sequence 2 |Af(k)|/N that we will denote as |A(k)|. With this scaling, we can see that the height of each harmonic in the frequency domain corresponds to the amplitude of the waveform in the time domain. Knowing the frequency components of the acceleration signal |A(k)|, we compared them with the maximum acceleration that a constant displacement of the subpixel limit value (for each frequency) would produce. The displacement–acceleration relationship in frequency is:

(5) $$A_{px}\left(k\right)=\frac{\;{\left(2\pi k\right)}^{2}}{9.8}\;dsubpixel.$$

We observed that at low frequencies, it is difficult to observe whether these frequencies exceeded the subpixel value, and more importantly, to have a qualitative idea of the displacement. We can get more detail at low frequencies by representing displacements instead of accelerations. For this, the maximum subpixel displacement will be constant in frequency and we will have to transform the accelerations to their corresponding displacements according to the function:

(6) $$D\left(k\right)=\frac{\left|A\left(k\right)\right|}{{\left(2\pi k\right)}^{2}}\;9.8.$$

It has been considered that when representing the discrete vectors, we join the values of the sequence to give a continuous appearance to the representation, so it is chosen to rename the independent variable and use f instead of k, as shown in the initial presentation, this being the one usually used in representations in continuous frequency, resulting in

(7) $$\left|A\left(f\right)\right|=\frac{2}{N}\left|Af\left(f\right)\right|,\;\;A_{px}\left(f\right)=\frac{{\left(2\pi f\right)}^{2}}{9.8}dsubpixel,\;\;D\left(f\right)=\frac{9.8}{{\left(2\pi f\right)}^{2}}\left|A\left(f\right)\right|.$$

**Figure 4:**
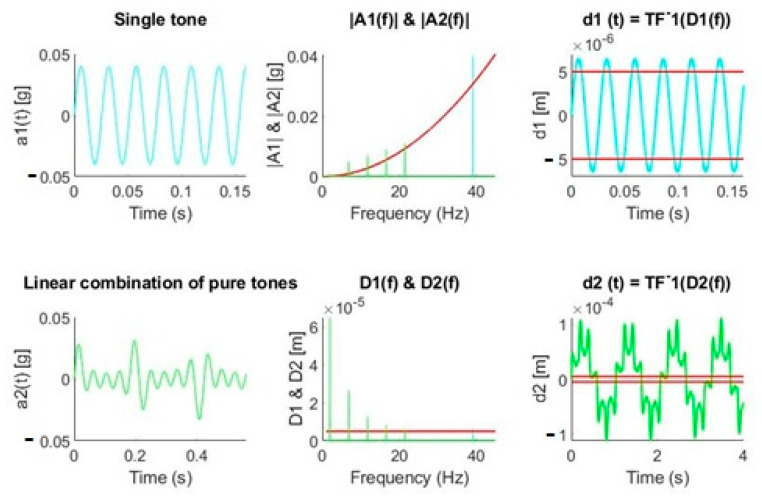
Acceleration-displacement conversion examples. Acceleration: ai(*t*): time domain, Ai(*f*): frequency domain. Displacement: di(*t*): time domain, Di(*f*): frequency domain. Single tone (cyan) and multi tone (green). Red line establishes the maximum value of acceleration or its corresponding displacement. TF^-^1 refers to the inverse Fourier Transform.

Figure 4 shows the process described for two cases of signals made up by pure tones. From the signal a_1_(*t*), we obtain the amplitude of such tone through the FFT, A_1_(*f*), and we represent it both in the acceleration and displacement graphs according to the expressions described above. Once the displacement produced by such tone has been obtained, we can visualize the instants of time in which the subpixel limit is exceeded in the temporal reconstruction. The signal a_2_(*t*), formed by the sum of tones of different amplitudes and frequencies, is converted to a frequency signal A_2_(*f*), and it is also drawn in the acceleration and displacement graphs, respectively. Subsequently, it is reconstructed, without taking into account lags, to the estimated displacement signal that would produce the initial acceleration a_2_(*t*).

From the examples presented, we can conclude that low accelerations in the range of m*g* at frequencies of a few hertz affect much more than higher accelerations at higher frequencies for a given displacement. The results are in line with the work of White [29], since he determined that high frequencies could not be associated with large displacements. This fact makes it necessary to achieve a good spectral representation of frequencies in the range of 1 to 10 Hz, which becomes difficult, as will be demonstrated in the following picture, if our signal processing is not capable of eliminating the DC level.

**Figure 5:**
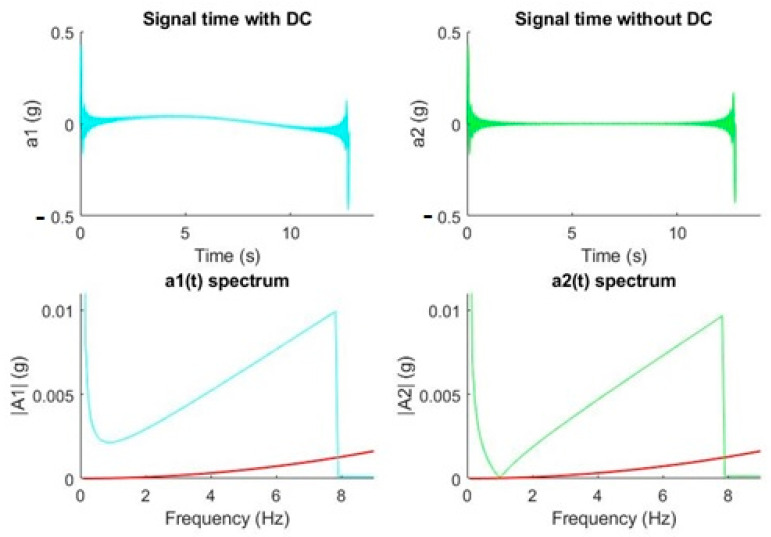
DC elimination effect on a synthesized signal. The red line establishes the maximum value of the acceleration.

To show the aforementioned idea, we proposed synthesizing a multi tonal signal consisting of 100 very close harmonics, 0.079 Hz apart from each other, distributed between 0 and 7.9 Hz. The amplitude of the harmonics increased in the form of a ramp reaching a maximum value of 0.01 *g* in 7.9 Hz. Figure 5 shows how a signal over time with a DC component does not reflect the original synthesized signal (continuous tones at each multiple of the spectral resolution from 0 to 7.9 Hz and increasing amplitudes from 0 to 0.01 *g*). However, if we eliminate this component as well as possible, the result is that the resulting spectrum fits what is expected. We realize that, for frequencies above 1 Hz, it is necessary to eliminate the DC component to be able to synthesize an acceptable version (in green) of the former multi tone signal. Below 1 Hz, not even the elimination of the DC component serves as a strategy: it was observed that for frequencies less than 1 Hz, the spectrum values are triggered because of the division by a squared frequency that tends to zero. This led us to have to discard the frequency components lower than 1 Hz.

## 4. Experimental Results

We developed four different experiments, showing typical movements performed by the T80 telescope, with the accelerometer configuration shown above. Acceleration was measured by three ultra-low frequency Wilcoxon 731A accelerometers placed in a plane parallel to the CCD. Then, the DC component was removed from the measured signals. The acceleration (and its corresponding displacement) were compared with a theoretical limit in order to verify that the vibration does not result in an unacceptable displacement.

The results were obtained from the remote laboratory, as explained in Section 2. All the experiments carried out at the telescope with a tracking movement in different configurations are explained below. It is necessary to note that the vibrations are greater in the positioning phase (tracking) than in the following phase (slewing). Although in these first data captures in the telescope we focused our measurements on tracking situations, in the future, we must focus our investigations on the measurement of vibration in the slewing phases. In any case, we analyzed the tracking movements to characterized in future works the time we must wait to take images in the tracking–slewing transition. The experiments showed the tracking movements of the actuator in charge of acting on the declination angle, the ascension movement as well as the set of actuators working at the same time.

### 4.1. First Experiment. Drift in the Decline Movements

**Figure 6:**
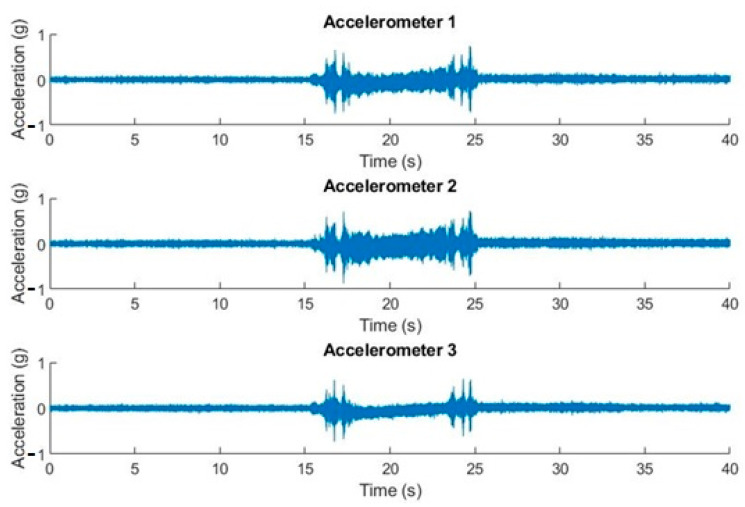
Acceleration in real time for the three accelerometers. Off-line graphic representation.

The conditions of the experiment shown in Figure 6 are declination movement from 0° to 40°, with the straight ascension angle fixed at 0°. The duration of the recording was 60 s and the sample rate = 10,240 samples/s. The sequence belongs to a T80 parking position tracking movement with a single actuator movement.

We can observe, on the same time scale and with simultaneous measurements, the state at rest and the movements produced by acceleration and deceleration. When the telescope goes into tracking mode for its positioning, stronger accelerations occur.

**Figure 7:**
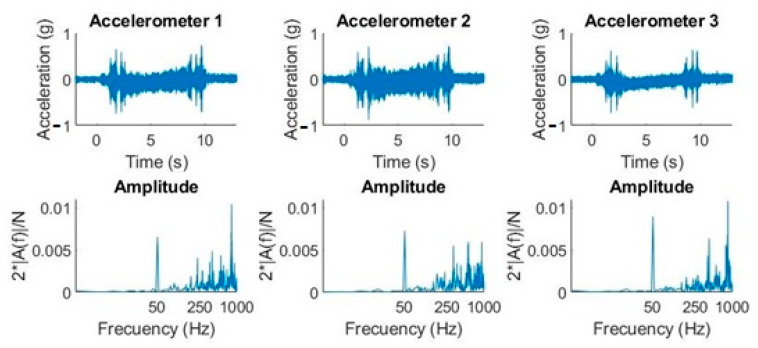
Graphs (acceleration-time and amplitude-frequency) with the three accelerometers in a declination movement from 0° to 40° and fixed straight ascension at 0°.

Figure 7 shows how the effect of electrical network (50 Hz) noise coupling had a significant influence in the frequency domain, but not in the time domain. The most notable collected amplitudes were in the neighborhood of 250 Hz, 400 Hz, 550 Hz, and 830 Hz, respectively.

### 4.2. Second Experiment. Straight Ascension Movement (from 0° to 40°) and Constant Decline 80°

The next experiment consisted of a straight ascension movement from 0° to 40° while the declination angle was constant 80°. The recording duration was 60 s and the sample rate = 10,240 samples/s. The sequence belonged to a tracking movement with a single actuator movement.

**Figure 8:**
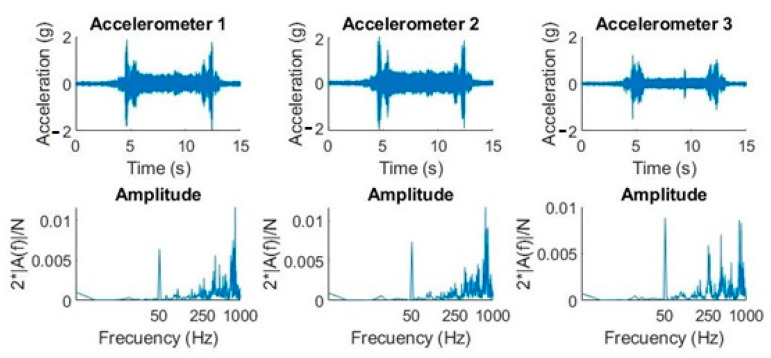
Graphs (acceleration-time and amplitude-frequency) with the three accelerometers in a straight ascension movement at 40° and declination at 0°.

If we compare the waveforms of the two movements (Figure 7 vs. Figure 8), we can observe that the amplitudes were very different between them. If we focus on accelerometer 1, for example, we observed that the declination amplitudes were below 1 *g* (Figure 7), so the maximum acceleration mechanically allowed by the CCD is satisfied. In contrast, in the straight ascension movement, the amplitudes reached approximately 2 *g* in the time domain (Figure 8), but if we pay attention to the theoretical quantification of the amplitude of the harmonics, we can observe how the largest amplitudes were at a high frequency. This is why, taking into account Figure 4, we can determine that it will not produce displacements greater than 5 µm.

### 4.3. Third Experiment. DC Filtering

**Figure 9:**
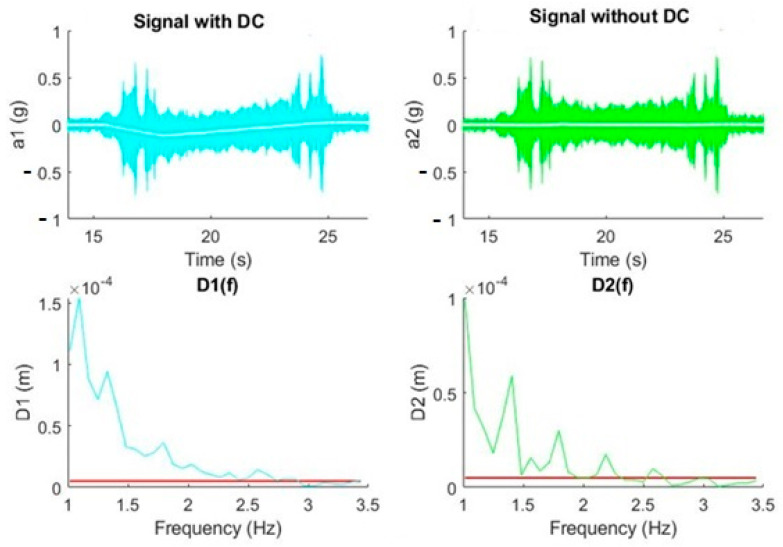
DC removal effect on a real signal (declination movement from 0° to 40°). The red line in the graphs establishes the maximum value of the displacement.

In this experiment, a real signal produced by a declination movement from 0° to 40° was analyzed. The DC component was eliminated in the time domain, validating the expected results according to the theoretical experiment in Figure 5. It was observed how the DC elimination decreased the power of the signal at frequencies between 1 Hz and 2 Hz, and therefore the displacement (see Figure 9).

**Figure 10:**
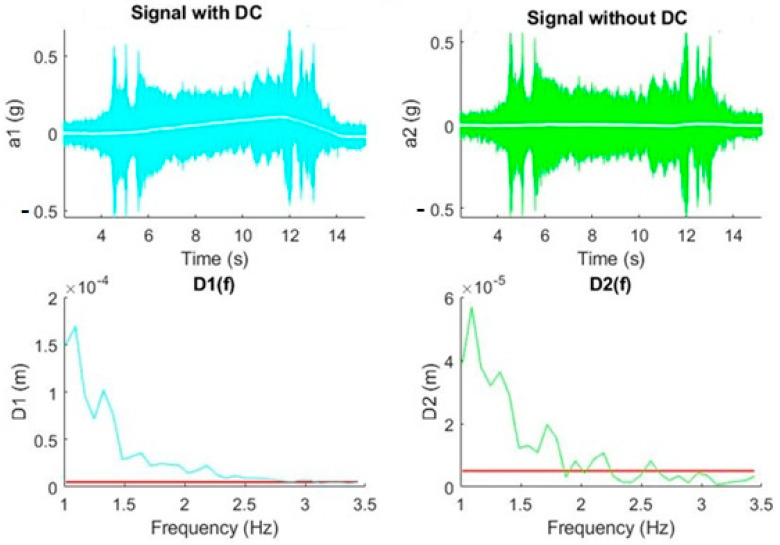
DC removal effect on a real signal (declination movement from 40° to 0°). The red line in the graphs establishes the maximum value of the displacement.

Figure 10 shows the same effect, an attenuation in power and consequently in displacement, when there is an inverse movement of declination (from 40° to 0°).

**Figure 11:**
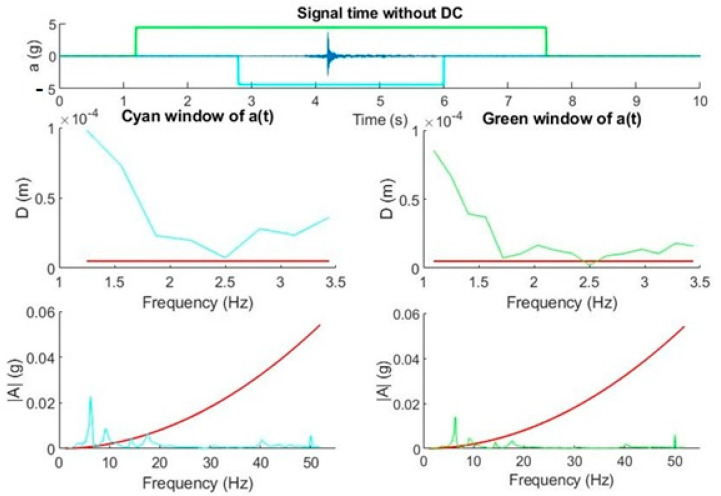
Graphs (acceleration-frequency and displacement-frequency). Straight ascension movement with abrupt movement of short duration. Influence of the windowing on the estimation of the characteristic frequency component of the signal. Visualization of maximum displacements. The red line in the graphs establishes the maximum value of the displacement and acceleration, respectively.

We developed an application that allowed us to monitor the frequency components present in the time intervals of an acceleration signal. The time intervals or windows can be chosen in such a way that the number of samples taken is a power of 2 to optimize the FFT. The application settings allow for setting the number of windows (1 to 4), their size, and the speed at which they should be scrolled, which will be the same for all windows. For each window, the program calculates A(*f*) and D(*f*) and displays it together with the corresponding maximum shift (in red color). All windows traverse the signal at the same speed, so they are centered. This makes it possible to contrast the effect of the size of the window. That is, if the signal of interest is present during the entire time of the window, the amplitude of the frequency components will be independent of the size of the window. In contrast, if the signal of interest lasts less than the window, the characteristic frequency components of the window will decrease as the window duration increases. In both cases, the spectral resolution decreases as the window decreases. Figure 11 shows the described effect.

We concluded that the characteristic frequency component of the signal to be measured was around 7 Hz and its duration was less than those belonging to the two windows, since it is the one that decreases when the size of the window increases. On the other hand, the lower frequency components (around 2 Hz) were present in the duration of the two windows since they varied very little. In this case, we can get closer to the characteristic amplitude of this signal by reducing the size of the window, with the limitation that the spectral resolution is somewhat less than 7 Hz.

### 4.4. Fourth Experiment. Coherence Analysis

**Figure 12:**
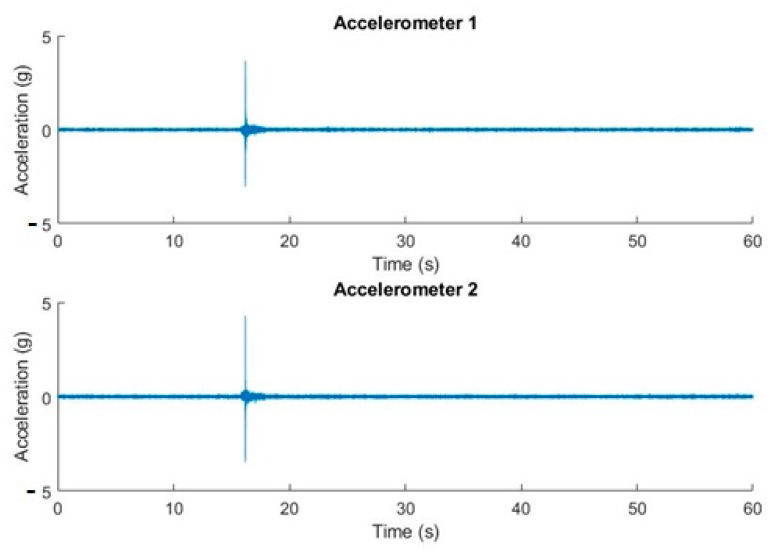
Waveform of channels 1 and 2. Straight ascension movement with vibration peak.

On the other hand, the coherence function is very useful to assess whether a vibration measured at a certain point may be the cause of a displacement at a second point, generally at a critical location. It is a powerful tool for optimizing sensor and actuator positions in vibration control systems. It can also be used simply as a similarity meter. Figure 12 shows the waveform of channels 1 and 2 of a straight ascension motion experiment where a problem occurs that causes a vibration peak of up to 4 *g*.

**Figure 13:**
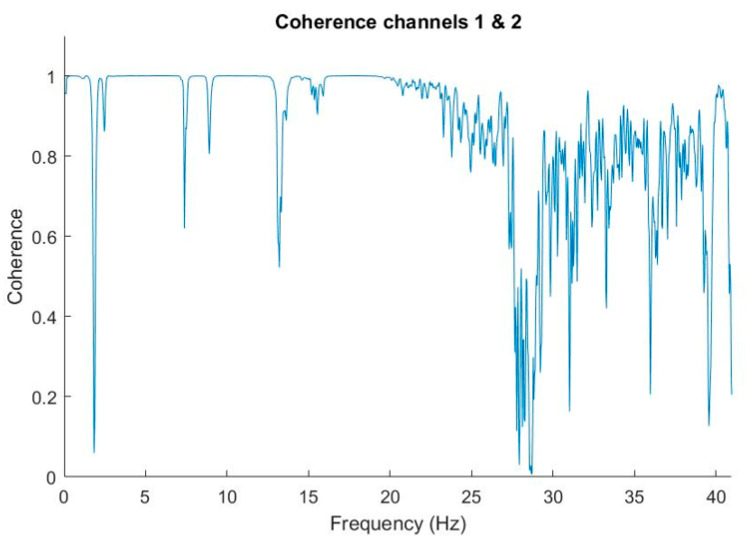
Coherence analysis between channels 1 and 2.

Finally, Figure 13 also shows the coherence between both channels in a very low frequency range. The coherence had a deep notch at 2 Hz and a wider one in the neighborhood of 27–28 Hz.

## 5. Discussion and Conclusions

The system developed in this study allowed for real-time monitoring of the displacements that occur in the CCD of the T80 telescope in order to determine whether or not the captured data are suitable for further processing. The necessary algorithms have also been developed to detect excessive displacements in the CCD of the T80 telescope and generate alarms that can be managed by the telescope operator. At the same time, the software generates log files with the time instant where there has been any problem due to vibrations.

From the experiments carried out, we might conclude that images should be rejected: during tracking movements of the telescope, during a transitory time (of around 10 s) after every tracking movement, or during short periods of time while slewing movements. Although the nature of these events is unpredictable, the continuous monitoring of vibrations and the subsequent signal processing techniques applied allowed us to obtain the exact duration of these events. This information is recorded in a file attached to the captured images. With that information, the telescope operator will determine the validity of the images with the data provided, according to the instants in which the vibrations have occurred, together with their duration. Therefore, image post-processing will avoid the affected images taken in those time instants. The software developed will also be used to detect possible electromechanical failures or those caused by weather phenomena.

The so developed monitoring remote system overcomes the access problems related to the location of large telescopes, which are normally located in high mountain areas where access is complicated due to both the orography and the weather conditions. This fact adds a notable improvement with respect to the studies found in the literature.

The study has shown that vibrations at low frequencies produce displacements greater than those allowed in the CCD, as we analyzed in the theoretical foundations in Section 3. Values of *mg* with frequencies in the range of a few hertz cause displacements that exceed the limit of 5 µm. We also observed, as expected due to the nature of the sensors used (piezoelectric type), a coupling of the electrical network signal (50 Hz) that appears in the frequency domain. The experiments carried out allowed us to observe the vibration that the T80Cam received at the location of the accelerometers on the *Z*-axis in the same plane as the CCD. This was possible using three accelerometers jointly placed in the filter box connected to a data acquisition card and remote transmission in real time from the JAO to the remote laboratory. The monitoring of the signals in real time has allowed us to observe, in the time domain, the maximum amplitudes that can affect the CCD and the mechanical structure.

The coherence analysis between the different accelerometers showed that the similarity in a wide range of frequencies was high. This implies that the positioning of an accelerometer in any of the corners of the filter box of the JPCam is sufficient to represent the vibrations that occur throughout the plane. This result will allow us, in the future, to use an accelerometer at a point in the current plane of the filter box and parallel to the CCD, to carry out the coherence analysis in the actuators and the structure. The latter analysis will allow us to determine the origin of the vibration, its amplitude, and determine the absorption of the vibration by the structure.

The results of our work are in line with that carried out by Trigona, Andró, and Baglio [15] at the “Serra la Nave” Observatory. They observed acceleration peaks near 5.5 Hz with amplitudes of 0.1*g*, which, as in our case, affected the quality of the image. They used a triaxial structure to measure accelerations in the three axes of the plane, not so in our case, due to the decompensation that would occur in the instrumentation. We used three accelerometers screwed on the *Z*-axis of the filter box, distributing the weight, and covering different measurement points on the same axis.

The problem of low frequencies was also evident in the Subaru Telescope [8] and the TMT telescope [14], in the latter with frequencies between 5 Hz and 20 Hz. They focused their study on the altazimuth motor gear as opposed to our study where we measured all vibrations that reached the instrumentation. The study on vibrations carried out by White [29] established the low frequencies as a dangerous range due to the displacement that it produces with low amplitudes, which supports the experiments.

The study carried out at the Daniel K. Inouye Telescope [12] was similar to our research regarding the use of low-frequency accelerometers. In this case, they were looking for shifts in the signal noise. Using spectral estimation, the measured noise ended up being vibrations from nearby traffic, machine shops, and other industrial plants. They also generated code to obtain coherence between accelerometers. The purpose was to validate the source of the disturbances at specific points on the mirrors without the noise produced from workshops or traffic. In our case, we used a coherence analysis to validate the position of the three accelerometers, although we intend to use this tool in the future to detect the origin of the vibrations.

As future work, we intend to extrapolate our analysis using less invasive technologies immune to electromagnetic noise such as fiber-optic-based technology. In addition to the advantages of electromagnetic immunity, the sensors have much smaller mechanical characteristics. These fiber-optic-based sensors are three times smaller than those used in this study and had a weight of 50 g, in contrast to the 750 g of those used in the present study. This will allow the placement of sensors on a triaxial structure without compromising the mechanical stability of the telescope. The coherence analysis software developed in this work will help us to carry out a complete vibration study in the T250 telescope and its JPCam instrumentation. Fiber optic-based technology (interrogator and accelerometers) will be used for this analysis. This telescope has just been assembled and has a 2.50-m primary mirror and the second largest camera in the world.

## Figures and Tables

**Table 1:** Wilcoxon 731A accelerometer characteristics.

Description	Wilcoxom 731A Accelerometer
Sensibility, ±10%, 25 °C	10 V/*g*
Frequency response	±10% 0.10–300 Hz ±3 dB 0.05–450 Hz
Resonance frequency	750 Hz
Temperature range	−10 °C to +65 °C
Vibration limit	10 *g* peak
Sensing element design	PZT ceramic/flexure
Weight	760 g

**Table 2:** NI 9148 Ethernet expansion chassis.

Description	NI 9148 Ethernet Expansion Chassis
Network interface	10BaseT and 100BaseTX Ethernet
Compatibility	IEEE 802.3
Communication rates	10 Mbps, 100 Mbps, auto-negotiated
Communication rates	10 Mbps, 100 Mbps, auto-negotiated

**Table 3:** NI 9234 DAQ.

Description	NI 9234
Number of channels	4 analog input channel
ADC resolution	24 bits
Type of ADC	Delta-Sigma (with analog prefiltering)
Sampling mode	Simultaneous
Data rate range	51.2 kS/s
Input range	±5 V
